# Knowledge, attitudes and practices in tamarind (*Tamarindus indica* L) use and conservation in Eastern Uganda

**DOI:** 10.1186/s13002-016-0133-8

**Published:** 2017-01-21

**Authors:** Esther Ebifa-Othieno, Antony Mugisha, Philip Nyeko, John David Kabasa

**Affiliations:** 1 Department of Geography & Social Studies, Kyambogo University, PO Box 1, Kyambogo, Kampala, Uganda; 2 College of Veterinary Medicine, Animal Resources and Biosecurity, Makerere University, PO Box 7062, Kampala, Uganda; 3 Department of Forestry, Bio-diversity and Tourism, College of Agricultural and Environmental Sciences, Makerere University, PO Box 7062, Kampala, Uganda

**Keywords:** *Tamarindus indica* L, Indigenous knowledge, Conservation, Uganda

## Abstract

**Background:**

*Tamarindus indica* L is one of the indigenous fruit tree species that traditionally contributes to food security and ecosystem stability in sub-Saharan Africa. We hypothesized that the indigenous people of Eastern Uganda have used *T. indica* for generations and developed practices that promote its conservation and therefore we expected that they possess an elaborate indigenous knowledge (IK) system and that most of them have planted the species. The aim of this study was to assess the influence of IK, attitudes and practices on the use and conservation of *T. indica*.

**Methods:**

A cross sectional survey was conducted in two districts purposively selected from the *T. indica* natural range. Focus group discussions, key informant interviews, semi-structured interviews and observation were used to collect data. Data was processed using qualitative analytical methods.

**Results:**

*Tamarindus indica* was highly valued by the majority of the population. *Tamarindus indica* was used for food, medicinal, cultural, social, environmental amelioration and income generation purposes. The population possessed a high level of IK about *T. indica* evidenced by 18 categories of uses and multiple modes of use. Fruit pulp was the most commonly used tamarind product. Relative frequency of citation of the different uses provides insight into usage levels and IK possessed. The communities’ food and medicinal uses concurred with scientific reports of health benefits of consuming *T. indica*. Approximately half of respondents had *T. indica* on their compounds or homegardens (53%). Fifty two percent of the tamarind population was self-propagated, 45% were planted while the propagation history for the remaining 3% was not known. Constraints towards planting *T. indica* included limited land, long maturation period and low monetary value. Fifty three percent of those who were growing *T. indica* did not carry out any silvicultural practices. The majority of *T. indica* encountered (87%) was intercropped with other crops or trees. Several beliefs and taboos regarding *T. indica* persist.

**Conclusion:**

*Tamarindus indica* has a high use value in the study area evidenced by multiple uses. Indigenous knowledge and uses concurred with scientifically proven nutritional and medicinal attributes of *T. indica* in literature which is significant given current trends towards affordable functional foods. The high level of IK has not translated into high rates of planting *T. indica*. There is need to encourage value-addition so as to maximize *T. indica* benefits and enhance conservation.

## Background

Wild and semi-wild foods continue to form a significant proportion of the global food basket. Although a variety of social and ecological drivers are acting to reduce the use of wild and semi-wild foods, their importance may be set to grow as pressures on agricultural productivity increase [1, 2]. Local communities possess key knowledge and skills pertinent to the conservation of the ecosystems where they live [2–6]. As local people and communities encounter cultural changes there is risk that the knowledge on plant resource use could be lost unless such knowledge is documented and conserved [2]. Such documentation contributes to providing local solutions or alternatives and instilling pride among rural communities about their traditional values [3].

Tamarind (*Tamarindus indica* L: Leguminosae), is an indigenous fruit tree of the tropics reported to be underutilised worldwide [7, 8]. Tamarind grows into a large evergreen tree, 20–30 m tall [9, 10]. *Tamarindus indica* possesses great potential to address various nutritional, health, socioeconomic and environmental constraints [7, 11, 12]. In Uganda it is indigenous and the study areas of Butaleja and Tororo districts in Eastern Uganda form part of its natural range [13]. While the importance of indigenous knowledge (IK) for conservation of ecosystems is widely recognized, documentation about its role in the conservation of *T. indica* in Uganda is very scanty. *Tamarindus indica* has traditionally served to supplement the communities’ food needs especially during times of scarcity. Although *T. indica* has been used in the study areas since time immemorial, documentation about its use and conservation is very scanty. According to Gadgil et al. [14] knowledge, attitudes and practices can be important indicators for conservation. Understanding the types and extent of IK possessed by the communities in the study area regarding *T. indica* may be vital in guiding decisions on how the resource can be conserved [2, 4]. Documenting the knowledge, attitudes and practices associated with *T. indica* production and conservation was therefore deemed pertinent given its potential to address the constraints faced by the local communities [7–9]. We hypothesized that the indigenous people of Eastern Uganda have used *T. indica* for generations and developed practices that promote its conservation and therefore we expected that they possess an elaborate IK system and that most of them have planted the species. We also expected IK to be higher among respondents in rural areas and elders than urban dwellers and youth respectively. The study aimed at assessing the influence of IK, attitudes and practices on the use and conservation of *T. indica*. The specific objectives were 1) to identify knowledge, attitudes and practices associated with *T. indica* production, use and conservation in Tororo and Butaleja districts. 2) To assess how knowledge, attitudes and practices have influenced *T. indica* conservation.

## Methods

### Study area

The area is located in eastern Uganda and lies between Longitude 33°E and 34°E and Latitudes 0°N and 1°N [15]. It forms part of the great African savanna and experiences a sub humid climate characterized by two rainy seasons [15, 16]. It lies at an altitude of between 1097 and 1219 m above sea level [16]. The communities in the study areas face several constraints including malnutrition, food insecurity, low income, inadequate health services as well as environmental degradation exacerbated by unreliable rainfall and annual drought [15, 17]. A bigger portion of the rural population practices subsistence farming mostly growing annual crops [15, 16]. Poverty is rampant [17].

### Study design

#### Selection of sites and respondents

**Fig. 1:**
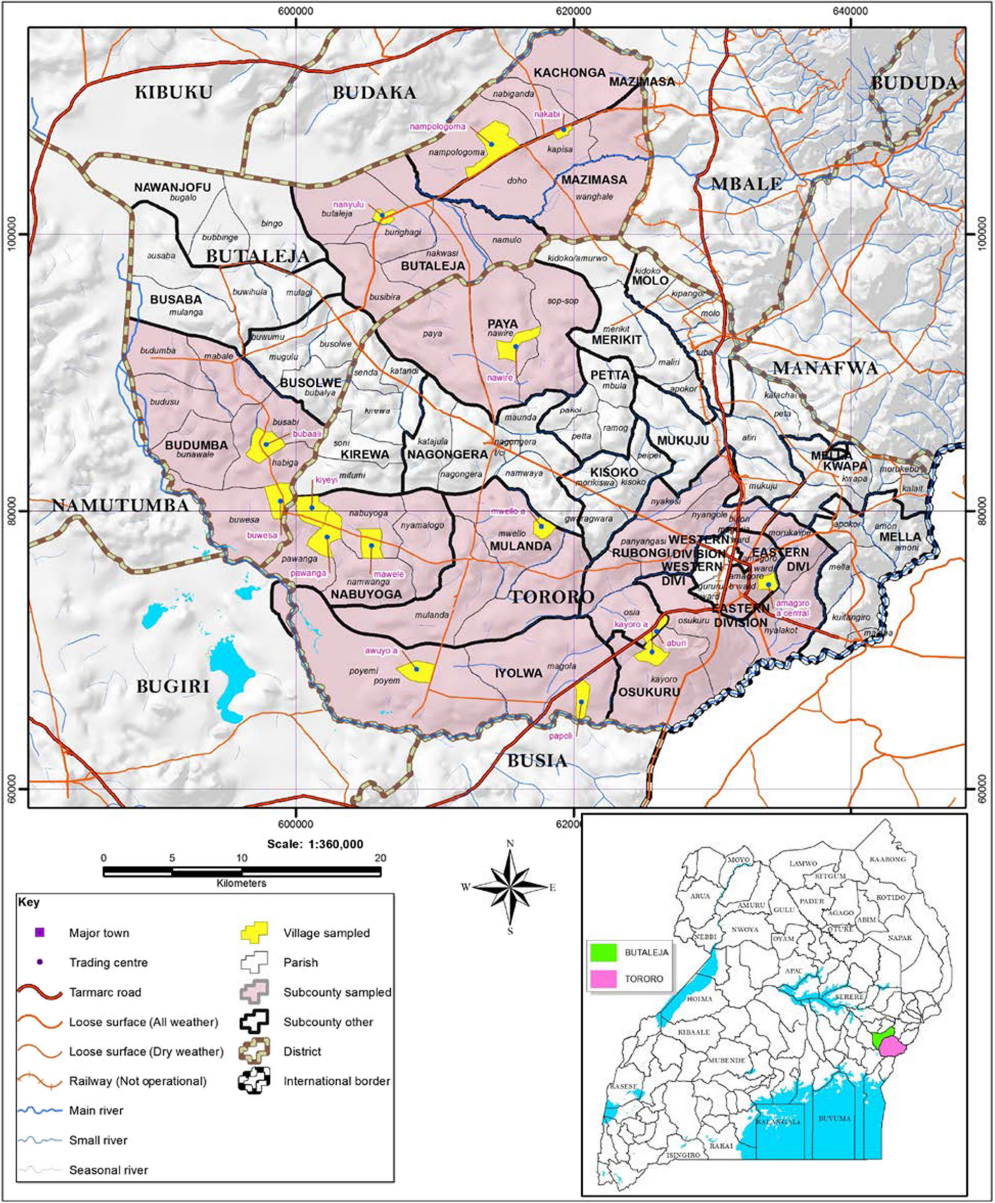
Location of study sites in Tororo and Butaleja districts, Eastern Uganda. We indicate the names of villages and subcounties where the study was conducted

A cross-sectional survey was carried out between October 2010 and February 2011 in Tororo and Butaleja districts, purposively selected on the basis of being among Ugandan districts within the *T. indica* natural range [13] and with a high poverty index [17, 18]. Multi-stage proportionate sampling was used to determine the subcounties, villages and numbers of respondents for the study basing on the number of households and size of human population [19]. Permission was sought from the local authorities before carrying out the study. Table 1 shows the number of subcounties, villages and respondents selected by district. Figure 1 shows the location of study sites.

**Table 1:** Sampled subcounties, villages and respondents by district

	Tororo district	Butaleja district	Total
Population^a^	379,399	157,489	536,888
Number of households^a^	80,334	31,949	112,283
Subcounties^a^	17	7	24
Subcounties sampled	8	4	12
Villages sampled	10	5	15
Semi-structured interviews	Males = 32 Females = 11	Males = 13 Females = 4	Males = 45 Females = 15
Key informant interviews	8	4	12
Focus group discussions	4	2	6

^a^population census figures [19]

Six focus group discussions, 12 key informant interviews and 60 semi-structured interviews were used to collect information about knowledge, beliefs, attitudes and practices [20–23] with regard to *T. indica*. Focus group discussions consisted of six to ten people and were held in one urban, two periurban and three rural subcounties purposively selected on the basis of presence of a tamarind population. Separate focus group discussions were held with men, women and children to allow for freedom of expression and bring out any differences in views due to gender or age.

Sixty households in total were selected for semi-structured interviews, a minimum of four and a maximum of nine households per subcounty. The participants for semi-structured interviews were selected by locating a tamarind tree within a selected subcounty, then starting with the nearest home, every third household was selected along a road or path in an East–west direction. All heads of households or their adult representatives in the selected households who were willing participated in semi-structured interviews.

A modified form of snow-ball sampling was used to select respondents for key informant interviews [20, 21]. Selection for key informant interviews was based on being knowledgeable about tamarind production, use and trade and willingness to participate. Only one key informant was selected per subcounty. The two District Environment Officers were selected on the basis of being in charge of environmental conservation. Ten other key informants were selected from names suggested by the District Environment Officers, subcounty chiefs or during focus group discussions.

Participants in focus group discussions, semi-structured interviews and key informant interviews were required to respond to several questions which covered the following themes: location of *T. indica*, uses, propagation, planting of *T. indica*, where *T. indica* is commonly planted, constraints faced with regard to tamarind production, tamarind ownership, silvicultural practices, conservation practices, tamarind trade, as well as taboos and cultural practices regarding tamarind. Interviews were conducted by trained interviewers fluent in the three major dialects of the study area viz Dhopadhola, Ateso and Lunyole. Oral informed consent/assent was obtained before participation.

#### Sampling of *Tamarindus indica* and associated crops/trees

Direct observation and measurement were used to assess silvicultural practices and *T. indica*/crop combinations. Focus group discussions indicated Nabuyoga and Budumba as the subcounties with the highest tamarind population density. A quadrat measuring 100 by 100 m was laid in each subcounty [22]. All *T. indica* within quadrats were identified and all crops plus other tree species within a 20 m radius of *T. indica* recorded. Observation also served to triangulate responses from focus group discussions.

#### Data handling and analysis

Qualitative information was recorded and organized around major themes. Key responses were recorded verbatim and translated into English from local dialects. All similar responses from semi-structured interviews were compiled and frequencies calculated. Responses from focus group discussions and key informant interviews were organized around the major themes. Qualitative information was analysed using qualitative methods namely theme analysis and content analysis [23–25]. Quantitative data were processed using MS Excel computer programme. Quantitative data on key variables was analysed and expressed using descriptive statistics (frequencies and percentages). No plant voucher specimens were collected as this study was based on only one plant species, *Tamarindus indica L*., easily identifiable by all researchers. Results of tamarind population studies will be published elsewhere.

## Results

### *Tamarindus indica* indigenous knowledge and uses

*Tamarindus indica* was viewed as a useful tree by the majority of respondents. *Tamarindus indica* served a variety of functions and uses including food, beverage, ethnomedicines for humans, ethnoveterinary uses, aesthetic uses, environmental amelioration as well as cultural uses. *Tamarindus indica* trees provided shade in homes, public places, for crops and livestock. Details of the various uses and mode of use of *T. indica* are summarized in Table 2.

**Table 2:** Uses of *Tamarindus indica,* parts used and mode of use

Uses	Part used	Mode of preparation and use
Food	Fruit	Mature ripe fruit of the sweet variety eaten as a snack. Immature green pods are eaten fresh or boiled with porridge to give it a sour taste. Pulp of mature ripe fruit added during preparation of porridge and millet bread to give it a sharp taste. Pulp concentrate is boiled to make a thick paste eaten as sauce especially during drought. Tender leaves cooked and eaten as a vegetable.
Beverage	Fruit	Husks are removed from mature ripe fruit which is then soaked in cold water. Seeds and fibres are then separated from the pulp to make a concentrate which is diluted to make a cold beverage popular with all ages. Sugar or honey may be added to enhance taste.
Spice/seasoning	Fruit	Pulp of ripe fruit boiled with dried potato chips ‘*amukeke*’(At.) adds flavor and preserves consistency. Pulp concentrate added to sauces such as meat to enhance taste.
Preservative	Fruit	Pulp added during preparation of millet bread preserves it for several weeks. Pulp added to sauces such as meat keeps it fresh for a longer period.
Income	Tree	Entire tree sold especially to limestone kiln operators to earn income.
	Fruit﻿	Fruit sold to earn income.
Fuel	Trunk	Trunk and large branches used to make charcoal. Makes excellent fuelwood for firing bricks and limestone kilns.
	Branches	Small branches lopped off during pruning or complete harvest are used for firewood.
mulch	Leaves	Leaves spread in gardens as mulch
construction	Branches	Straight portions are used in house construction
Tools and utensils	Trunk and branches	Small stems and branches are used to make clubs and tool handles for hoes, axes and pangas Trunk is chiseled to make utensils such as mortars, pestles
Aesthetic and recreation	Seeds	Seeds are used as counters in traditional board games such as ‘*omweso*’ (lus)
	Tree	Trees add beauty to homes and provide shade in homesteads and other compounds thus improving the ambience
Socio-cultural	Tree	Due to the cool shade and lack of parasites, large tamarind trees are favourite venues for village meetings, markets and places of worship. Large tamarind trees are used as polling stations during elections. Due to their longevity, tamarind trees serve as key landmarks and are often used as reference points and boundary markers during land demarcation between neighbours.
Education	Seeds	Seeds are used as learning aids during arithmetic lessons for beginners
Personal hygiene	branches	Ends of small branches are cut and the ends chewed to make durable toothbrushes
Ethnoveterinary uses	Leaves	Freshly picked mature leaves are crushed in water and decoction used to treat livestock diseases such as ‘*kawali*’(dhop.)
Ethnomedicine for humans	Leaves	Freshly picked mature leaves crushed in water then filtrate mixed in porridge to treat ‘*kawali*’(dhop.) (smallpox) Leaves crushed in water and drunk to treat abdominal upsets. Decoction of stem bark used to treat abdominal upsets in humans.
	Fruit	Pulp diluted to make a cold beverage given especially to those undergoing stress such as pregnant women, convalescents and those returning from war. Fruit pulp concentrate used to treat constipation
Shade	Tree	Provides shade for livestock, in homesteads, on compounds and for travellers along roads
Windbreak	Tree	Windbreak for houses and crops
Support	Tree	Trees used to support climbing plants including passion fruit, yams and oyster nuts locally known *kulekula*[lus] or *Onjwege* [dhop.] Straight portions of branches used to support banana stems
Feed	Leaves	Fresh leaves are fed to domestic animals such as goats.

dialects: at = ateso, dhop = dhopadhola, lus = lusoga, lug = luganda

*Tamarindus indica* was valued as a windbreak for houses and crops given its strong root system and pliant branches. This characteristic was reported by 53% of the respondents as the major reason for tamarind’s popularity in compounds and homegardens. The vicinity of a tamarind tree was a preferred site for house construction as expressed in the words of one of the respondents: ‘*building your house close to a tamarind tree is advantageous since you are assured of a windbreak. Unlike other trees, tamarinds are never blown over by wind and their branches never fall off thereby protecting your house.*’

The most commonly used tamarind part was the fruit (100%). Table 3 is a summary of frequency citation of *T. indica* uses. All the respondents mentioned that they made a popular beverage from *T. indica* fruit pulp (Table 3). Tamarind fruit was eaten as snack by almost all the respondents (98.33% citation). Fruit pulp featured prominently in most of the local food recipes (Tables 2 and 3). Ethnomedicinal uses for humans and ethnoveterinary uses had the lowest percent citations (13 and 15% respectively).

**Table 3:** Frequency citations of *Tamarindus indica* uses

Use	Tamarind part(s) used	Butaleja (*n* = 17)	Tororo (*n* = 43)	Total FC^*^ per use	RFC^**^	Percentage citation
Beverage	Fruit pulp	17	43	60	1	100
Food (snack)	Fruit	17	42	59	0.98	98.33
Windbreak	Trees	12	41	53	0.88	88.30
Oral hygiene (toothbrushes)	Branches	11	42	53	0.88	88.30
Shade	Trees	15	37	52	0.87	86.70
Flavouring	Fruitpulp	11	24	45	0.75	75
Aesthetic/recreation purposes	Trees	12	32	44	0.73	73
Firewood and charcoal	Trunk, branches	2	36	38	0.63	63
Teaching/learning aid	Seeds	0	28	28	0.47	46.70
Food preservative	Fruitpulp	4	20	24	0.40	40
Source of income	Trunk, fruit, branches	2	21	23	0.38	38.30
Construction	Trunk, branches	2	19	21	0.35	35
Feed for livestock	Leaves	1	20	21	0.35	35
Tool handles and clubs	Branches, trunk	2	15	17	0.28	28
Mulch	Leaves	2	14	16	0.27	26.70
Support for plants	Trees	2	9	11	0.18	18
Ethnoveterinary uses	Leaves, stembark, rootbark	2	7	9	0.15	15
Ethnomedicine for humans	Leaves, stembark, rootbark	3	5	8	0.13	13

FC*Frequency of citation

RFC** Relative frequency of citation = Frequency of citation (FC) divided by number of respondents (*n* = 60)

Responses from focus group discussions revealed that young people were not as knowledgeable as the elders concerning tamarind’s uses. Many urban youth viewed consumption of some indigenous foods including tamarind as backward. Youth in rural areas were more interested in earning an income from tamarind products. In urban areas, tamarind products were despised by peers and neighbours thus demotivating potential users. Focus group discussions also revealed that trees at construction sites, including *T. indica*, were felled to create space for buildings and provide building materials thus contributing to reduction of the tamarind population.

### Tamarind locations

*Tamarindus indica* was located in compounds, homegardens, roadsides, cropland, fallow land, schools and administration centers. Approximately half of the respondents had *T. indica* on their compounds or in homegardens (53%).

*Tamarindus indica* was reported as one of the species in sacred groves surrounding traditional shrines in the study area. Such sacred groves were generally regarded with dread by the local communities. The research team did not access *T. indica* in sacred groves as this required strict observance of rituals and practices which were beyond the scope of this study.

### *Tamarindus indica* propagation and silvicultural practices

**Fig. 2:**
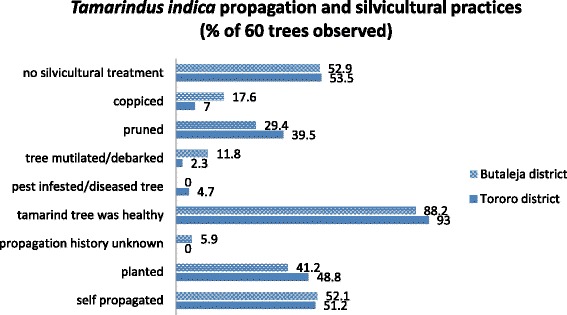
Tamarind propagation and silvicultural practices

Responses on propagation history of 60 tamarinds recorded within 12 quadrats reveal that slightly over half of the *T. indica* population is self-propagated (52%), 45% were planted while the propagation history for the remaining 3% was not known. Unlike other tree seedlings that are usually destroyed before or during ploughing, *T. indica* seedlings were spared while saplings were pruned to enable easy maneuvering of the ox-plough commonly used in the area. The common practice was to spare *T. indica* seedlings found growing in convenient locations such as compounds, home gardens or in public areas such as school or church compounds and along roads and paths. Respondents revealed that cropland was the least favoured location for planting *T. indica* because it competed for limited space with foodcrops. Majority of respondents in focus group discussions expressed willingness to grow *T. indica* if assured of financial benefit. Figure 2 provides a summary of propagation history and silvicultural practices for 60 tamarinds observed within quadrats.

The majority of *T. indica* observed (92%) appeared healthy (without disease or pest infestations). Incidences of damages on the trees were minimal (5.0%). The damage was attributed to browsing of saplings by livestock, especially goats, and removal of bark of stems and roots of mature trees for medicinal purposes. Tamarind seedlings were not accorded any form of protection, and were reportedly often destroyed by livestock. Approximately half of the respondents (53%) did not add fertilizer or carry out any other silvicultural practices on tamarinds. Thirty seven percent reported pruning *T. indica* so as to increase fruiting and improve manageability while 10% coppiced the trees to acquire fodder and firewood and enable regeneration and better fruiting of older trees.

### Crops and trees grown with *Tamarindus indica*

**Fig. 3:**
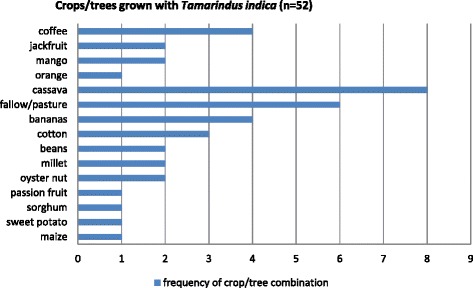
*Tamarindus indica*/crop combinations in Eastern Uganda

*Tamarindus indica* was found both in the wild and on cultivated land. Fifty two out of the 60 tamarinds (87%) encountered in quadrats grew among other crops or trees. Figure 3 shows details of 15 observed *T. indica*/other trees/crop combinations and their frequencies. Cassava was the most common crop found growing with *T. indica*. Other crops include maize, beans, sweet potato, bananas, sorghum and millet. *Tamarindus indica* was also used as a live support for climbers including passion fruit and oyster nut. Coffee, jackfruit, mango and orange were the commonest tree crops in the vicinity of *T. indica*. Although focus group discussions indicated that *T. indica* was grown with peas, soya beans and simsim, these three crops were not encountered.

### Ownership of tamarind trees

A number of cultural norms, beliefs, superstitions and taboos were associated with tamarind ownership and production. Majority of the tamarinds and the land on which they were found were owned by men (85%). Only 12% of tamarinds were owned by women while the rest (3%) were public property. Planting of *T. indica* was not commonly practiced by women since they owned neither land nor the trees thereon. Table 4 outlines the gender roles, beliefs and taboos associated with *T. indica* ownership, production and trade.

**Table 4:** Gender roles, beliefs and taboos associated with *T.indica* ownership, production and trade by district

District	Tororo			Butaleja		
Gender/age group	M	W	C	M	W	C
1. Who owns *T. indica* trees in this area?	√	√	×	√	×	×
2. Who commonly plants *T. indica*?	√	√	√	×	×	×
3. Who is allowed to harvest tamarind products?	√	√	√	√	√	√
4. Who processes *T. indica*?	√	√	√	√	√	√
5. Who commonly sells *T. indica*?	√	√	√	×	×	×
6. Who is allowed to consume tamarind products?	√	√	√	√	√	√
7. Who commonly buys tamarind fruit at markets for consumption?	×	√	×	×	√	√
8. Who commonly buys tamarind at farms for reselling?	√	×	×	×	×	×
9. Are there any taboos associated with *T. indica* production or consumption?	×	√	×	×	×	×

*M* men, *W* women, *C* children

### Harvesting, processing and consumption of *Tamarindus indica*

In the study areas *T. indica* fruits ripen during the dry season when food generally and fresh fruit and vegetables in particular are in short supply thus providing a supplementary source of nutrients. Furthermore *T. indica* is easily accessible to all community members. Harvesting was mostly done by young men and children. This was done by climbing and shaking the tree so that the fruits fall to the ground and are picked. Alternatively a long stick was used to remove the fruits. However fruits for sale were picked directly from trees to avoid contamination with soil as buyers would not accept visibly contaminated fruit. Alternatively, the higher branches were accessed using a ladder. The bark was most often accessed from the ground. Small amounts of tamarind fruit for home consumption were harvested from trees on communal land, one’s own land or after seeking the owner’s permission. Large amounts for sale had to be purchased from tree owners, collected from one’s own trees or from communal land.

Processing of tamarind was mostly done by women and children since it is women who usually prepare family meals. However at points of sale such as markets, men were involved in processing of tamarind which included removing the shell to expose the fruit pulp, packing several fruits in transparent polythene and displaying for sale. In homes, the fruits were dehusked and soaked in water to soften. Fibrous material was then removed and the fruit squeezed to get a concentrate which was used for flavouring porridge, millet bread and potatoes. Alternatively the concentrate was diluted to make a cool refreshing beverage popular with all age groups. Sugar or honey was often added to enhance taste. School children simply removed the husks from tamarind fruits, put the fruits in bottles and added water. The bottle was then shaken to make a type of ‘tamarindade’ which was enjoyed immensely. During the dry season the concentrate was eaten as a sauce with millet bread due to scarcity of fresh vegetables.

Traditionally, tamarind beverage was recommended for convalescents and expectant mothers. Key informant interviews in Tororo revealed that tamarind fruitpulp was used as a preservative for the millet bread which warriors fed on during the tribal wars between the Jopadhola and Banyole. Tamarind fruit beverage was also commonly given to rejuvenate those returning from war.

### Beliefs, taboos and superstitions associated with *Tamarindus indica*

Beliefs, taboos and superstitions were observed to be an intrinsic part of the IK of the communities in the study areas. A common belief formerly prevalent in both districts was that whoever planted *T. indica* would die before tasting its fruit. In the words of one respondent: ‘*Planting a tamarind tree is pointless since one cannot live long enough to eat its fruit. You can only plant for your children and grandchildren.*’ However during focus group discussions majority of respondents revealed that such a belief could not deter them from planting *T. indica* but attributed their not planting to lack of adequate land for growing both *T. indica* and other crops. The words of one 80-year old widow were insightful: ‘*My husband planted a tamarind seedling on our compound hoping to only benefit from its shade. However he ate of its fruit for more than 5 years before his death. It has the sweetest fruit of all the three tamarind trees on our land. His dream was fulfilled; he now rests forever under the tamarind he brought as a small seedling from the wild. As for me, his children and grandchildren, we continue to enjoy its fruit*’. Traditionally, the local inhabitants bury their dead on the compound or in the homestead. In this way the memory of their dead relatives is honoured and preserved as the graves are never far away from the living. It was observed that most of the *T. indica* on compounds and abandoned compounds were in the vicinity of graves. This probably points to the close relationship between culture and *T. indica* in these communities.

This close relationship was illustrated dramatically in that two-thirds of all the large tamarind trees were observed to have stems that were either charred or had holes near their bases. Interviews revealed that during funerals, meals were prepared under tamarind trees since they are usually free from parasites. Being hardy, *T. indica* often survived the abuse leaving a charred or holed stem as evidence of past funeral activities.

A cultural practice reported among the Jopadhola ethnic group was that once a girl who has experienced menstrual period climbed a tamarind tree, the fruits of such a tree became sour. Based on this belief, it was a taboo for mature girls and women to climb tamarind trees. No specific taboos concerning harvesting *T. indica* were reported in Butaleja district except for *T. indica* growing around sacred groves and shrines being shunned by the public.

### Trade in *Tamarindus indica* products

Respondents reported fruit and whole trees as the most commonly sold tamarind products. Trees were mainly sold to operators of limestone kilns and brick makers. Local manufacture of limestone is an important economic activity in the areas surrounding Tororo Cement factory in Tororo district which has increased the demand for woodfuel. Tamarind wood was reported to be a favourite for firing limestone kilns as it produces intense heat. Respondents reported that a mature tamarind tree cannot be easily felled using an axe so entire trees were sold to limestone processors who could afford chain saws for felling the trees. In the words of one respondent: ‘*Only the rich can afford to fell a tamarind tree so we sell at a giveaway price. Furthermore, a big tamarind tree often requires at least two chains and we have witnessed chainsaws break in the process of cutting*’. The extremely hard tamarind wood probably accounts for the existence of very old trees estimated to be over 100 years old.

Respondents in Tororo district reported selling tamarind fruit with its shells still intact for the equivalent of half a United States dollar for smaller heaps (≤1 kg) and up to one dollar for a basinful (3–8 kg). Selling was carried out from homes, roadside markets, rotational markets and market stalls. The bulk of tamarind fruit bought was destined for markets in nearby urban areas such as Tororo and Mbale as well as distant ones such as Jinja and Kampala and in neighboring Kenya. Tamarind fruit was sold by both men and women. Respondents in Butaleja district did not report selling any tamarind product.

## Discussion

The majority of respondents viewed *T. indica* as a valuable resource. This is in agreement with Bourou et al. [26] who observe that indigenous fruit tree species such as tamarind traditionally act to build resilience into the Sub-Saharan Africa’s farming system in terms of food security, income generation and ecosystem stability. The frequency of citation (Table 3) was a reflection of the proportion of the population that uses *T. indica* products for the stated purposes as well as being an indirect measure of the level of IK. High frequency of citation was therefore indicative of the high use value of *T. indica.* This is in agreement with Havinga et al. [27] who report that *T. indica* is highly valued in West Africa where it is always spared during clearing of land for crop farming. Tamarind fruit beverage had the highest percent citation (100%) indicating it was consumed by all respondents. Ethnomedicinal uses for humans and ethnoveterinary uses had the lowest percent citations (13 and 15% respectively) probably because such knowledge is possessed by only a few people mostly elders, herbalists and traditional healers. Noss et al. [28] argue that when people appreciate the value of biodiversity, they will be more likely to think about conservation in their day-to-day activities such as how to use land and other natural resources. The selective sparing of *T. indica* seedlings points to the high value attached to it compared to other tree species in the study area. On the other hand focus group discussions revealed that *T. indica* was often felled to create space for buildings and provide building materials. This is a reflection of competing landuses especially in urban areas. Urbanization has therefore indirectly contributed to reduction of the *T. indica* population.

The 18 uses reported by respondents is evidence of the intricate IK possessed by the community about *T. indica*. Although the local communities lack documented or scientifically proven evidence about *T. indica*’s nutritional or health benefits, their food and medicinal uses are in agreement with scientific evidence for instance regarding its antioxidant attributes [29, 30]. The relevance of IK is further illustrated by the traditional practice of feeding tamarind fruit pulp to pregnant women and warriors to boost health and recovery. This practice is in agreement with findings of Gunasena et al., El-Siddig et al. and De Caluwé et al. who report *T. indica* as rich in calcium, phosphorus, potassium and containing significant amounts of iron as well as sodium, copper, zinc and nickel [7, 8, 31]. Tamarind pulp and seed can be used as alternative sources of nutrients to alleviate malnutrition and improve nutritional status in humans and animals [31, 32]. Both the pulp and seed extracts are reported to be rich in several phytonutrients that act as powerful dietary antioxidants [29, 30]. According to De Caluwé et al. [31], the leaves are a fair source of vitamin C and β-carotene and mineral content is high, particularly P, K, Ca and Mg. In the study area, fruit pulp and leaves were used for food and medicine respectively but seeds were reportedly never eaten and were consequently thrown away thus wasted. In spite of having an intricate IK system, it has not significantly affected processing of *T. indica* as no value-added products were encountered and *T. indica* remains underutilized.

Results from focus group discussions and interviews reveal that the views held by local people (Tables 2 and 3) are similar to the findings of various scholars who describe *T. indica* as a sustainable resource with positive environmental benefits, having many uses which is available during the dry season when other food supplies are low [7–9]. *Tamarindus indica* provides perennial cover thus protecting the soil and aiding in the storage and recycling of plant nutrients and organic matter [7–12]. Focus group discussions commended *T. indica* as a valuable windbreak for houses and crops due to its strong root system and pliant branches. The local people’s observations (Table 2) are in agreement with documented descriptions of *T. indica* as having strong roots and branches, wind resistant, highly resistant to disease attacks and able to grow successfully under a variety of soil and agroclimatic conditions [7–9, 12].

Several authors including El-Siddig et al., De Caluwé et al. and Havinga et al. [8, 27, 31] attest the potential of *T. indica* to boost nutrition, income generation and environmental conservation (Table 5). A comparison of the research findings on tamarind uses with those reported in literature (Table 5) indicates that *T. indica* is largely underutilised in the study area. This can be attributed to several factors including limited knowledge about its nutritional and medicinal potential plus lack of appropriate technology to enable processing of *T. indica* into useful products.

**Table 5:** Previous research findings on *Tamarindus indica* L uses and functions

Uses	Reference notes
food	Ripe fruit eaten as snack [8]. Unripe fruits (swells) are roasted on coals and eaten with wood ashes in the Bahamas [8]. Fruit pulp is an important ingredient in chutneys, sauces and confectionaries [7, 8, 31]. Seeds are peeled then roasted or boiled and eaten [11].
	Leaves and flowers eaten as vegetables or prepared in a variety of dishes. Pulp and leaves are used to make curries, salads, stews and soups in India and Zimbabwe [7, 8, 11, 12, 31].
Spice/seasoning	Tamarind juice is an important ingredient of barbecue sauces such as Worcestershire sauce [8]. The tender, immature, very sour pods are cooked as seasoning with rice, fish and meats in India [7]. The fruit pulp is used to give a sour taste to sorghum or millet porridge and bread in Uganda and the Sahel [11].
Beverage	Tamarind fruit pulp is sometimes combined with guava, papaya, banana or made into wine [31]. Sweetened drinks made from tamarind fruit pulp are popular in the tropics and are bottled in carbonated form in Guatemala, Mexico and Puerto Rico. Formulas for the commercial production of spiced tamarind beverages have been developed by technologists in India [7].
Environmental amelioration	*Tamarindus indica* can be planted as a shade. Suitable for wind and firebreak [9, 12]. Used in Nigeria in antidesertification programs. Very suitable for sequestering carbon from the atmosphere due to its longevity [11]. *Tamarindus indica* can withstand harsh environmental conditions such as prolonged drought. *Tamarindus indica* can be used to reclaim poor soils, degraded land, rocky terrain and recently salinized soils [7, 9].
medicinal	*Tamarindus indica* fruit pulp used in the treatment of a number of ailments including fevers, rheumatism, throat infections as well as possessing anti-fungal and anti-bacterial properties. Seeds are a valuable remedy in diarrhea and dysentery [31]. Fruit, bark and leaves are used to treat diarrhea and as a laxative in East and West Africa [27].
	Dietary antioxidants can be extracted from *T. indica* fruit pulp and seed. Pulp and seed can also serve as alternative source of nutrients to alleviate malnutrition [30].
	Fluorosis caused by water containing Fluorine can be effectively prevented by dietary inclusion of tamarind pulp [36, 37].
Industrial uses	Tamarind fruit shells carbon is a promising adsorbent for removing fluoride from groundwater [38].
	Dyes are obtained from the leaves and flowers [8].
	Tamarind seed is the key raw material for the manufacture of tamarind seed kernel powder (TKP), polysaccharide (jellose), adhesive and tannin. TKP is an important sizing material in textile, paper and jute industries [8]. Tamarind seed pectin can form gels over a wide pH range [31]. Ten percent TKP is used as binder in making sawdust fuel briquettes [38].
	Tamarind seed is a good source of protein and oil and steadily gaining importance as an alternative source of protein as it is rich in certain essential amino acids [8, 30, 32].
	Tamarind fruit pulp is an important natural source of tartaric acid [8]. Tartaric acid is added to other foods to give a sour taste, used as an antioxidant and in the preparation of copper (I) oxide [8].
Animal feed	Seeds are used to make livestock feed [38, 39].
Cosmetics	*Tamarindus indica* seeds are used as a base in cosmetics [38].
Aesthetic	*Tamarindus indica* is planted to beautify compounds and avenues [9].
Furniture and utencils	The very hard and durable wood is reported to be excellent for poles, timber, boat-building, toys, tool handles, turnery products, furniture, decorative panelling and general construction work [7, 9].
Fuel	*Tamarindus indica* wood gives off an intense heat approaching ≈5000 cal per kg thus valued for firing brick kilns [7].
Socio-cultural	*Tamarindus indica* is very suitable for resting and meetings due to the evergreen habit and the extending crown thus provides shade for both people and livestock [9]. Over-ripe fruits and roots mixed with sea salt are used to clean and brighten silver, copper and brass in India [8].
Religious	*Tamarindus indica* is believed to be sacred in some African and Indian tribes [7, 12]. Used to cleanse a new house in India by marrying *T. indica* to a mango tree [8].

The population possessed a vast amount of IK about *T. indica* uses (Table 3). This is in agreement with Shapi et al. [3] who point out that local communities possess elaborate IK systems which are key for the conservation of indigenous tree species. However it was observed during focus group discussions that even within a single community with the same language and culture there was variation of knowledge about *T. indica* between the youth and the older generation. Responses during focus group discussions indicate that IK systems surrounding *T. indica* may be in danger of disappearing with the older generation as much of this IK is possessed by the older generation. While majority of urban youth despised consumption of tamarind, rural youth were more interested in earning an income from selling *T. indica* products. However school age children in both rural and urban areas appreciated tamarind fruit. Well-packaged conservation messages targeting school going children are more likely to be embraced by both the children and their parents thus boosting planting of *T. indica*.

Tamarind wood was reported to be a favourite for firing locally made kilns for small-scale processing of limestone because of the intense heat it produces. This can be attributed to its high calorific value which is estimated at ≈5000 cal per kg [7, 9]. Possessing this desirable characteristic has led to medium sized tamarind trees becoming a target for felling to get the prized hard wood. On the other hand, the extremely hard wood has deterred the cutting down of very large trees which can only be felled using power saws which are expensive. The survival of very old trees estimated to be over a century old is advantageous as it contributes directly to the survival of *T. indica* populations in the area.

Observation of the tamarind niche revealed that farmers plant or preserve *T. indica* with other tree species. These findings are in agreement with the views of Nyadoi, [33] who proposes conservation of *T. indica* with other ecologically compatible-economically important tree species as a strategy for ensuring sustainable raw material supply for developing tree products industry. Eighty seven percent of *T. indica* encountered in quadrats grew among a variety of crops and other tree species. Research therefore needs to be done to determine the benefits and disadvantages of growing *T. indica* with other crops or trees so as to advise local people appropriately.

The presence of *T. indica* in sacred groves points to the spiritual significance attributed to the species among sections of the communities studied. Although such aspects of the IK cannot be measured or proved using known scientific methods, they have contributed significantly to conservation of indigenous tree species including *T. indica. Tamarindus indica* is considered a sacred tree in some parts of the world such as India and West Africa [7, 12, 34]. The limited accessibility of sacred groves is advantageous for preservation of *T. indica* in situ since trees therein are not usually cut down. These findings concur with Banana et al. [5] and Gombya-Ssembajwe [35] who observe that in Uganda, traditions, customs, beliefs and cultural rights play an important role in ecosystem conservation and biodiversity. The observation that most of the *T. indica* on homesteads and abandoned compounds was in the vicinity of graves also points to the close relationship between culture and *T. indica* in these communities.

## Conclusion and recommendations

The high value attached to *T. indica* has contributed to its continued existence in the study areas. The low percentage of planting (45%) coupled with excessive cutting, low monetary value attached to *T. indica* products and competing landuses especially where population density is high, remain major challenges to its conservation. Having an intricate IK has not translated into high rates of planting *T. indica* or other conservation practices.

Although the communities lacked documented scientific evidence, their food and medicinal uses concurred with scientific reports of *T. indica* health promoting attributes. That IK and uses in the study area concurred with scientifically proven nutritional and medicinal attributes of *T. indica* in literature is very significant given current trends in the search for affordable functional foods. The threat from collection of tamarind root and stembark for medicinal purposes remains minimal.

There is need to increase overall tree conservation efforts since *T. indica* grows together with other tree species. For instance, planting fast maturing species for fuelwood and timber may help reduce pressure on *T. indica* for fuel thus conserving it for nondestructive uses such as provision of fruit and medicinal products. Efforts aimed at food security need to be aligned with environmental conservation to reinforce each other for effectiveness and maximum benefit to local communities.

The constraints reported with regard to planting *T. indica* need to be addressed so as to boost planting. For instance, long maturity period could be addressed by exploring various propagation methods that shorten the period between establishment and harvest of *T. indica*. There is also need to explore ways of increasing earnings from *T. indica*. Propagation of varieties with desirable characteristics for instance ‘sweet fruit’ or ‘high yielding’ need to be promoted. Concerns about limited land could be addressed through identifying potential locations for planting *T. indica* such as along boundaries, roads, compounds and public land. Further investigation is needed to elucidate the relationship between *T. indica* and surrounding plants.

Land and tree tenure arrangements that discourage women from planting *T. indica* need to be addressed. Empowering women to own land could be adopted as a long term strategy to promote planting of *T. indica* and other tree species for general environmental conservation. Sensitizing women about the nutritional benefits of various *T. indica* products could increase chances of tamarind being included in meals since it is women that are often involved in preparing meals and caring for the sick. Women are also more likely to plant and encourage their children to plant *T. indica* if knowledgeable about its nutritional and economic benefits.

The IK possessed by locals was shrouded in myths and superstitions. Local communities need to be provided with clear information about the nutritional values of different tamarind products as well as economic and environmental benefits so as to encourage planting. Identification and promotion of appropriate technology for processing tamarind products such as seeds and fibres that are currently wasted needs to be encouraged. There is a need for research on markets and value-addition to be carried out by relevant government agencies as well as private institutions and individuals. Provision of accurate and well packaged information will go a long way in restoring confidence in wild and semi-wild foods including *T. indica*.

## Abbreviations

FAO: Food and Agriculture Organization of the United Nations
IK: Indigenous Knowledge

## References

[1] Will M. Promoting value chains of neglected and underutilized species for pro-poor growth and biodiversity conservation. Guidelines and good practices. Global Facilitation Unit for Underutilized Species, Rome, Italy; 2008.

[2] Bharucha Z, Pretty J (2010). The roles and values of wild foods in agricultural systems. Phil Trans R Soc B.

[3] Shapi M, Cheikhyoussef A, Mumbengegwi DR, Matengu K, Van Kent A, Sifani J (2012). Evolution of data collection methods for indigenous knowledge systems at the Multidisciplinary Research Centre of the University of Namibia.

[4] Trosper RL, Parrotta JA, Parrotta JA, Trosper RL (2012). Introduction: the growing importance of traditional forest-related knowledge. (Chapter 1). Traditional forest-related knowledge: sustaining communities, ecosystems and biocultural diversity. World Forest Series vol 12.

[5] Banana A, Bahati J, Gombya-Ssembajjwe W, Vogt N. “Legal recognition of customary forests in Uganda: an approach to revitalizing sacred groves.” African sacred groves: ecological dynamics and social change. Sheridan MJ, Nyamweru C, editors. Oxford: James Currey; Athens: Ohio University Press; 2008. p. 195–206.

[6] Ebifa E. Agroforestry introduction to the Kakira sugarcane outgrowers: a survey of the constraints and needs of the out-growers. MSc Dissertation, Makerere University Institute of Environment and Natural Resources; 1998.

[7] Gunasena HPM, Hughes A. Tamarind. International Centre for Underutilised Crops, Southampton, UK; 2000.

[8] El-Siddig K, Gunasena HPM, Prasad BA, Pushpakumara DKNG, Raman KVR, Vijayanand P, Williams JT. Tamarind (*Tamarindus indica* L). Fruits for the Future 1 – Revised. International Centre for Underutilised Crops, Southampton, UK; 2006. http://www.icuc-iwmi.org/.

[9] Fichtl, R. The Tamarind. 2005. http://www.beesfordevelopment.org. Accessed 14 Dec 2008.

[10] California Rare Fruit Growers, Inc. (CRFG).Tamarind. 1996. http://www.crfg.org/pubs/ff.

[11] The National Academies Press (NAP). America’s National Research Council. ‘African fruits hold great potential to combat poverty’. 2008. p 158.

[12] Morton JF. Fruits of warm climates. Creative Resources Systems; 1987 p. 115–21.

[13] Katende AB, Birnie A, Tengnas B. Useful trees and shrubs for Uganda: identification, propagation and management for agricultural and pastoral communities. Regional Soil Conservation Unit. 2000. Technical handbook 10. Accessed from: www.worldagroforestry.org/downloads/publications/PDFS/b09383.pdf.

[14] Gadgil M, Berkes F, Folke C. Indigenous knowledge for biodiversity conservation. Ambio. 1993; 22:2/3 Biodiversity: Ecology, Economics, Policy. p. 151–6. http://www.jstor.org/stable/4314060. Accessed 8 June 2011.

[15] Government of Uganda. National Environment Management Authority (NEMA). TORORO District State of Environment Report. 1997.

[16] Uganda Districts Information Handbook. Expanded edition 2005–2006. Fountain Publishers.

[17] UNDP Uganda Human Development Report 2007. Rediscovering agriculture for human development. http://planipolis.iiep.unesco.org/upload/Uganda/Uganda_National_Human_Development_Report_2007.pdf.

[18] Government of Uganda. Status and future plans for food and nutrition security in Uganda. Office of the Prime Minister; 2011.

[19] Uganda Bureau of Statistics. 2002 Uganda Population and Housing Census Annex 3.

[20] Marshall M. Sampling for qualitative research. Family Practice. 1996;13:522–525. http://fampra.oxfordjournals.org/. 10.1093/fampra/13.6.522 9023528

[21] Gill P, Stewart K, Treasure E, Chadwick B (2008). Methods of data collection in qualitative research: interviews and focus groups. Br Dent J.

[22] Duchok R, Kent K, Khumbongmayum AD, Paul A, Khan ML (2005). Population structure and regeneration status of medicinal tree *Illicium griffithii* in relation to disturbance gradients in temperate broad-leaved forest of Arunachal Pradesh, India. Curr Sci.

[23] Taylor-Powell E, Renner M. Analyzing qualitative data. Madison: University ofWisconsin Extension; 2003. Available from: https://learningstore.uwex.edu/Assets/pdfs/G3658-12.pdf.

[24] Nalubega R, Nyanzi SA, Nakavuma JL, Kamatenesi-Mugisha M (2013). Ethnobotanical uses of Lantana trifolia L. and Sida cuneifolia Roxb. in Mukungwe and Wabinyonyi Sub-counties of Central Uganda. J Intercult Ethnopharmacol.

[25] Tardio J, Parrdo-De-Santayana M (2008). Cultural importance indices: a comparative analysis based on the useful wild plants of southern Cantabria (northern Spain). Econ Bot.

[26] Bourou S, Bowe C, Diouf M, Van Damme P. Ecological and human impacts on stand density and distribution of tamarind (*Tamarindus indica* L.) in Senegal. Afr J Ecol. 2012;50(3):253–380.

[27] Havinga RM, Hartl A, Putscher J, Prehsler S, Buchmann C, Vogl CR (2010). *Tamarindus indica* L. (Fabaceae): patterns of use in traditional African medicine. J Ethnopharmacol.

[28] Noss RF, Dobson AP, Baldwin R, Beier P, Davis CR, Dellasala DA, Francis J, Locke H, Nowak K, Lopez R, Reining C, Trombulak SC, Tabor G (2011). Editorial. Bolder thinking for conservation. Conserv Biol.

[29] Stangeland T, Remberg SF, Lye KA. Antioxidants in some Ugandan fruits. Afr J Ecol. 2007;45:29–30.

[30] Khairunnuur FA, Zulkhairi A, Azrina A, Moklas MAM, Khairullizam S, Zamree MS, Shahidan MA (2009). Nutritional composition, *in vitro* antioxidant activity and *Artemia salina* L. Lethality of pulp and seed of *Tamarindus indica* L. extracts. Mal J Nutr.

[31] De Caluwé E, Halamová K, Van Damme P. Tamarind (Tamarindus indica L.): a review of traditional uses, phytochemistry and pharmacology in African natural plant products: new discoveries and challenges in chemistry and quality. American Chemical Society; 2009: p. 85–110.

[32] Yusuf AA, Mofio BM, Ahmed AB. Proximate and mineral composition of *Tamarindus indica* Linn 1753 seeds. Sci World J. 2007;2(1). http://www.scienceworldjournal.com/.

[33] Nyadoi P. *Tamarindus indica* L. Genetic structure and niche ecology PhD Thesis, Makerere University. 2010.

[34] Fandohan AB, Assogbadjo AE, Sinsin B. Endogenous Knowledge on Tamarind (*Tamarindus indica* L.) in Nothern Benin. 2008. In Parotta JA, Oteng-Yeboa A, Cobbinah J (editors). Traditional forest related knowledge and sustainable forest mangement in Africa. Accra, Ghana. 2008. https://www.cabdirect.org/abstracts/20113396756.html.

[35] Gombya-Ssembajjwe W. Indigenous technical knowledge and forest management: a case study of sacred groves (Traditional Forest Reserves), Mpigi District, Uganda. 1997.

[36] Ekambaram P, Namitha T, Bhuvaneswari S, Aruljothi S, Vasanth D, Saravanakumar M. Therapeutic efficacy of *Tamarindus indica* L to protect against fluoride-induced oxidative stress in the liver of female rats. Fluoride.2010;43(2):134–40. http://www.fluorideresearch.org/432/files/FJ2010_v43_n2_p134-140.pdf.

[37] Dey S, Swamp D, Anju S, Ananya D (2011). In vivo efficacy of Tamarind (*Tamarindus indica* L) fruit extract on experimental fluoride exposure in rats. Res Vet Science.

[38] Bhattacharya S, Bal S, Mukherjee RK, Bhattacharya S (1993). Some physical and engineering Properties of tamarind (*Tamarindus indica* L.) seed. J Food Eng.

[39] Aengwanich W, Suttajit M, Srikhun T, Boonsorn T (2009). Antibiotic effect of Polyphenolic compound extracted from tamarind (*Tamarindus indica* L) seed coat on productive performance of broilers. Int J Poultry Sci.

